# Supplementary material for: Intrinsic Flexibility of the EMT Zeolite Framework under Pressure
Source: Molecules. 2019 Feb 12;24(3):641. doi: 10.3390/molecules24030641 (PMC6384661; doi:10.3390/molecules24030641)
Supplement: Supplementary file 1 [file molecules-24-00641-s001.pdf]

Article

Intrinsic Flexibility of the EMT Zeolite Framework under Pressure: Electronic supporting information

Antony Nearchou [1], Mero-Lee U. Cornelius [2], Jonathan M. Skelton [1,3], Zöe L. Jones [1], Andrew B. Cairns [4], Ines E. Collings [5], Paul R. Raithby [1], Stephen A. Wells [6] and Asel Sartbaeva [1,*]

1. Department of Chemistry, University of Bath, Claverton Down, Bath BA2 7AY, UK; a.nearchou@bath.ac.uk (A.N.); jonathan.skelton@manchester.ac.uk (J.M.S.); zlj20@bath.ac.uk (Z.L.J.); chsprr@bath.ac.uk (P.R.R.)
2. Department of Chemistry, University of the Western Cape, Bellville, Cape Town 7535, South Africa; meroleecornelius@gmail.com
3. School of Chemistry, University of Manchester, Oxford Road, Manchester M13 9PL, UK
4. Department of Materials, Imperial College London, Kensington, London SW7 2AZ, UK; a.cairns@imperial.ac.uk
5. European Synchrotron Radiation Facility, 71 avenue des Martyrs, 38000 Grenoble, France; ines.collings@esrf.fr
6. Department of Chemical Engineering, University of Bath, Claverton Down, Bath BA2 7AY, UK; saw42@bath.ac.uk

* Correspondence: a.sartbaeva@bath.ac.uk; Tel.: +44(0)-1225-385410

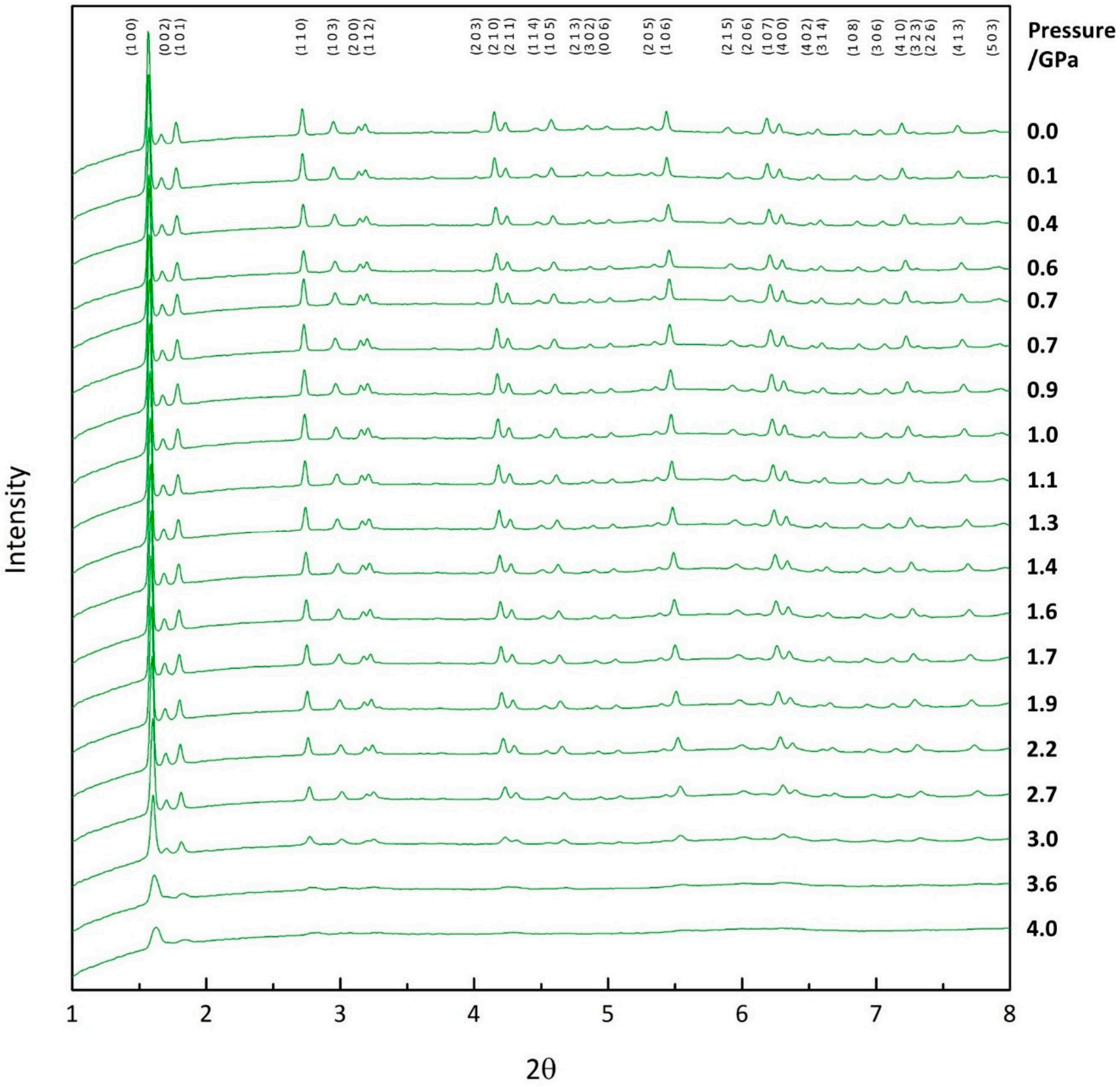

Figure S1: Powder X-ray diffraction patterns (2θ = 1-8°) of empty zeolite EMC-2 (EMT) as a function of pressure. Patterns were collected on the ID15B beamline at the ESRF, Grenoble, France, using Daphne 7373 oil as a non-penetrating pressure transmitting medium. Broadening of the Bragg diffraction peaks at higher pressures indicate substantial loss of crystallinity, i.e. pressure-induced amorphisation, particularly between 3.0-3.6 GPa.

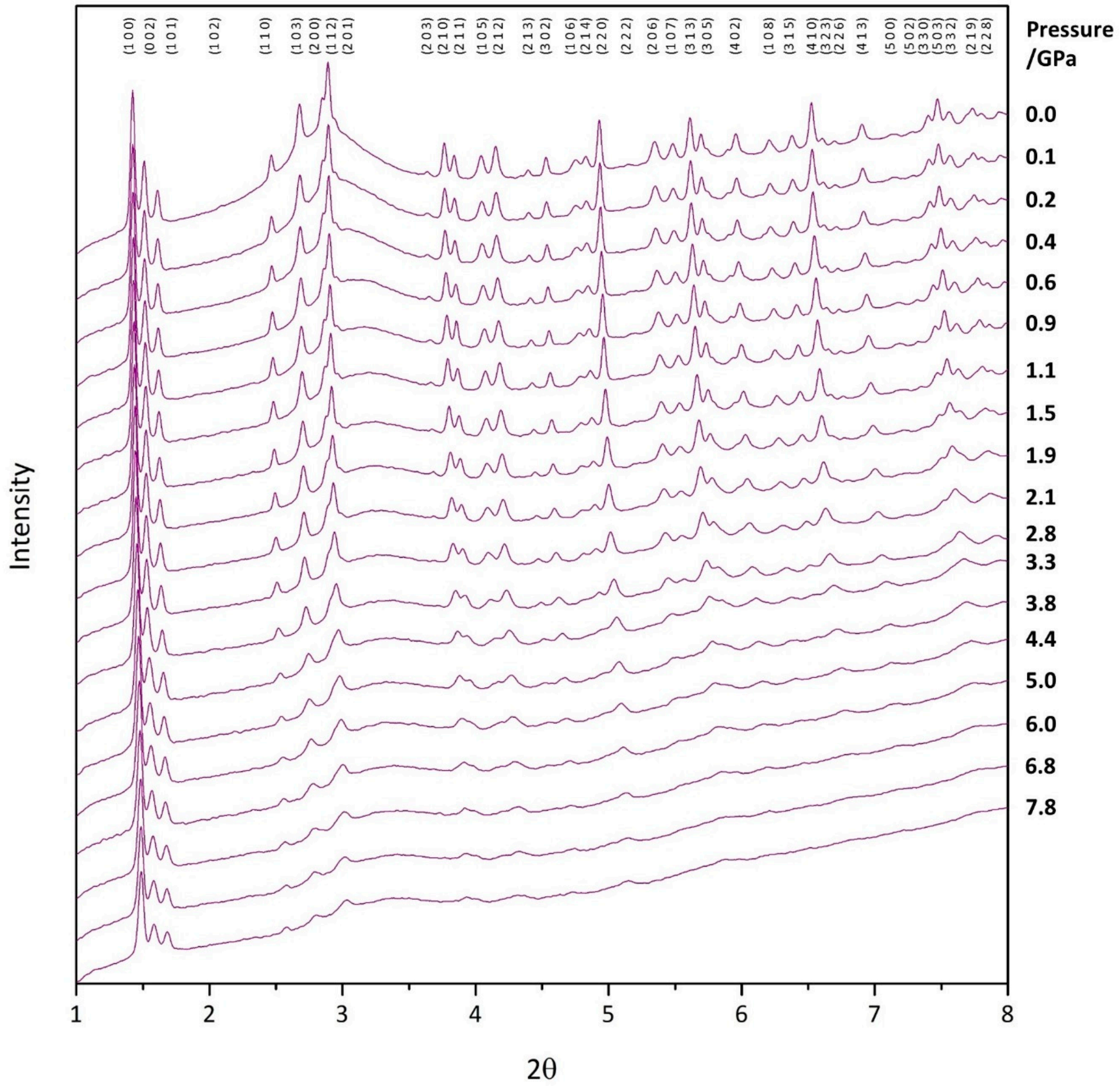

Figure S2: Powder X-ray diffraction patterns (2θ = 1-8°) of filled zeolite EMC-2 (EMT) as a function of pressure. Patterns were collected on the ID27 beamline at the ESRF, Grenoble, France, using silicone oil as a non-penetrating pressure transmitting medium. As for the empty framework (Figure S1), the patterns indicate substantial loss of crystallinity with pressure.

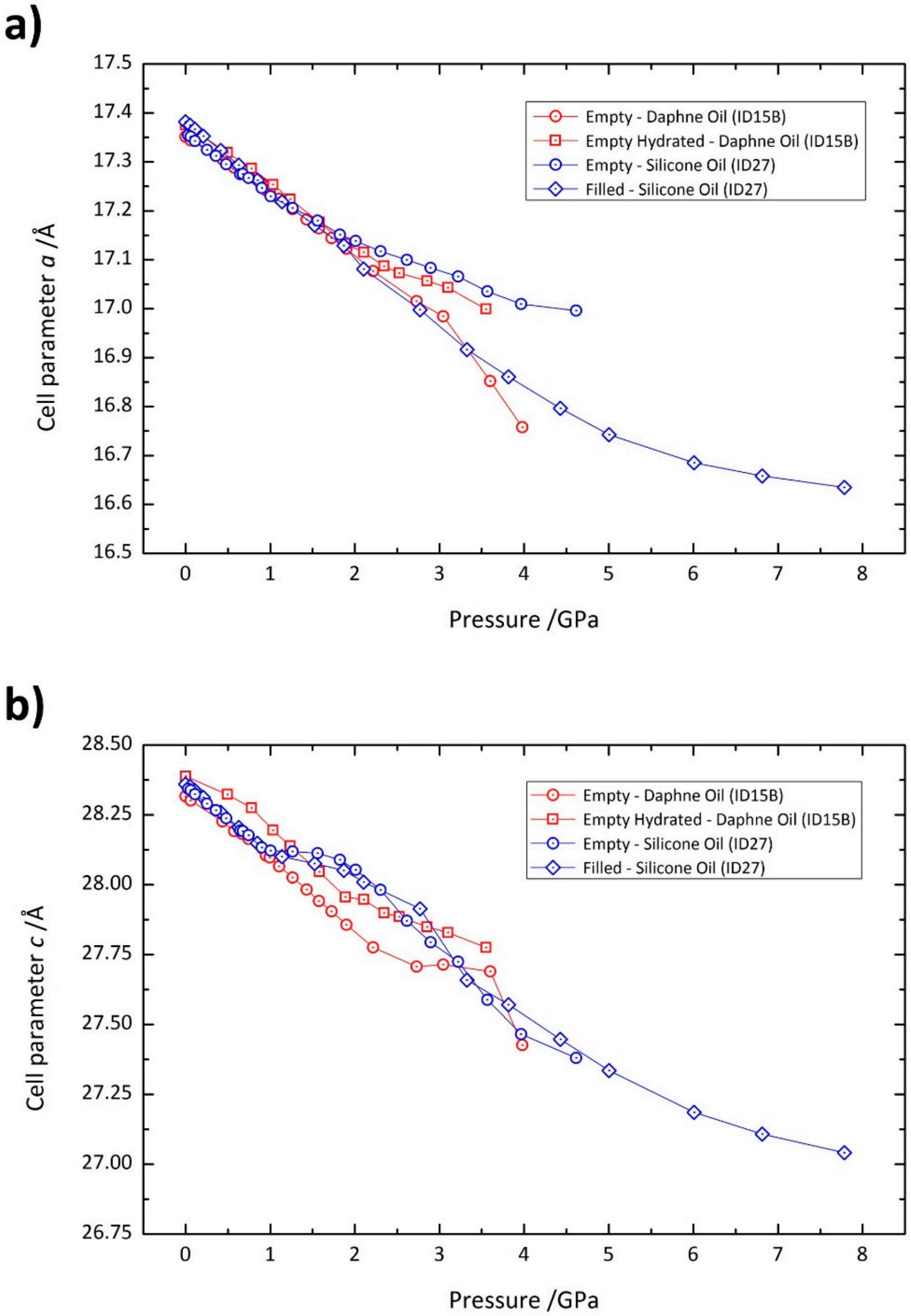

Figure S3: Unit cell contraction of zeolite EMC-2 (EMT) samples recorded under pressure. The variation in (a) cell parameter *a* and (b) cell parameter *c* as a function of pressure for various zeolite EMC-2 (EMT) sample measurements. Datapoints in red correspond to samples measured using Daphne 7373 oil (as non-penetrating pressure transmitting media) on beamline ID15B, and those in blue refer to samples measured using silicone oil on beamline ID27. Circles correspond to empty samples, squares to hydrated empty and diamonds to filled.

The hydrated sample has larger cell parameters due to the presence of the extra-framework water. These data demonstrate that the *a* parameter is consistent amongst all samples up to approximately 2.2 GPa, where the pressure transmitting media solidifies [62]. As for the *c* parameter, only the samples analysed using silicone oil on beamline ID15B show the anisotropic contraction in the 1-3 GPa range. This highlights that these observations are due to the breakdown of the silicone oil's hydrostatic behaviour [36,63].

a)

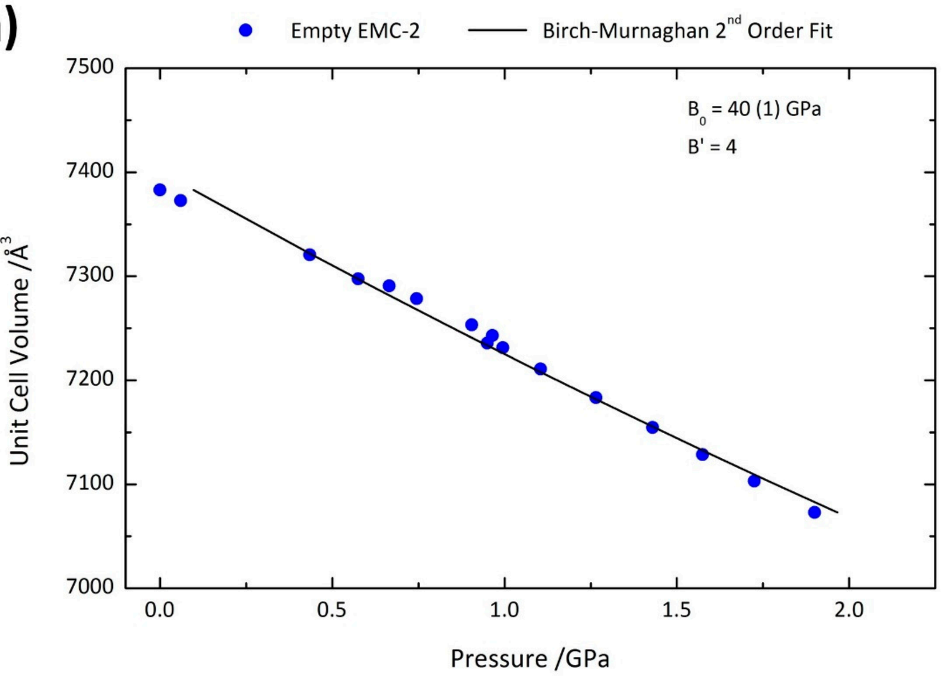

b)

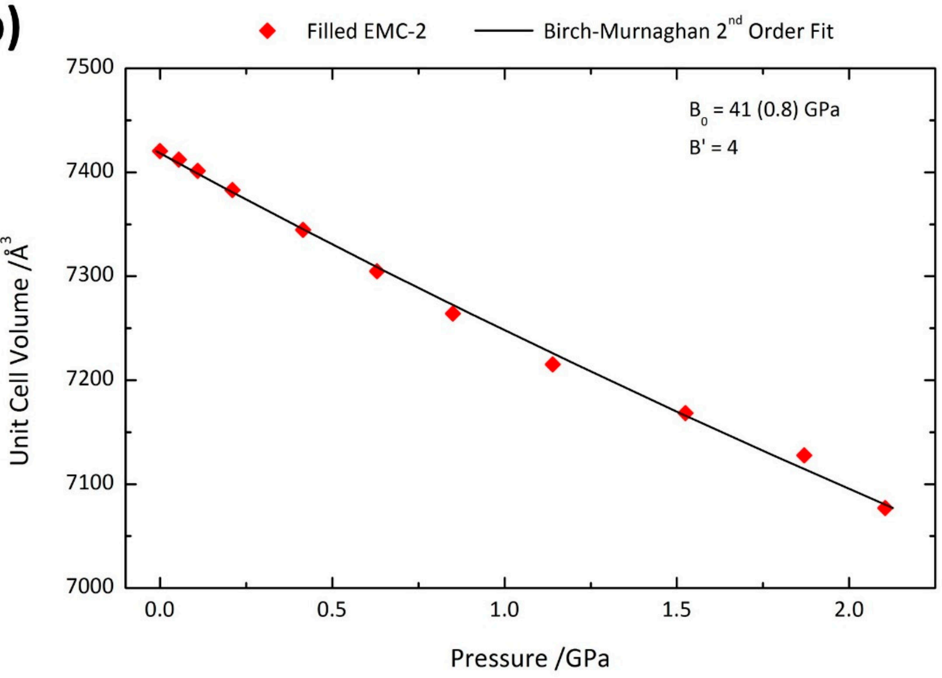

Figure S4: Unit cell volume contraction and Bulk Moduli for the zeolite EMC-2 (EMT) samples. The unit cell volume is shown as a function of pressure for the empty (a) and filled (b) zeolite EMC-2 (EMT) in the range of 0-2.2 GPa. The solid lines show fits to the 2nd-order Birch-Murnaghan equation, obtained using the PASCal web application [37], with fit parameters as marked.

Table S1: Optimised lattice parameters of faujasite (FAU) and EMC-2 (EMT) obtained with the PBEsol and PBEsol+D3 exchange-correlation functionals. Lattice parameters obtained with an implicit-solvent model used to mimic the dielectric environment of water (ε = 80.1) in the cages are also shown. % differences with respect to the experimental lattice parameters are given in parentheses. The parameters for FAU are given with respect to the conventional cubic unit cell.

	FAU		EMT		
XC Functional	a [Å]	V [Å^3]	a [Å]	c [Å]	V [Å^3]
PBEsol	24.466<br>(+0.50)	14,645<br>(+1.50)	17.309<br>(+0.54)	28.202<br>(+0.43)	7,317<br>(+1.52)
PBEsol (H_2O)	24.465<br>(+0.49)	14,644<br>(+1.49)	17.311<br>(+0.56)	28.186<br>(+0.37)	7,315<br>(+1.49)
PBEsol+D3	24.392<br>(+0.19)	14,512<br>(+0.58)	17.265<br>(+0.29)	28.114<br>(+0.11)	7,258<br>(+0.70)
PBEsol+D3 (H_2O)	24.409<br>(+0.26)	14,543<br>(+0.79)	17.272<br>(+0.33)	28.102<br>(+0.07)	7,260<br>(+0.74)

Table S2: Elastic-constant matrix of faujasite (FAU) calculated using the PBEsol+D3 functional. The calculated bulk and shear moduli, using the relations in Ref. [64], are 40 and 14.1 GPa respectively.

C_{ij} [GPa]	1	2	3	4	5	6
1	58.4	30.8	30.8	-	-	-
2	30.8	58.4	30.8	-	-	-
3	30.8	30.8	58.4	-	-	-
4	-	-	-	14.3	-	-
5	-	-	-	-	14.3	-
6	-	-	-	-	-	14.3

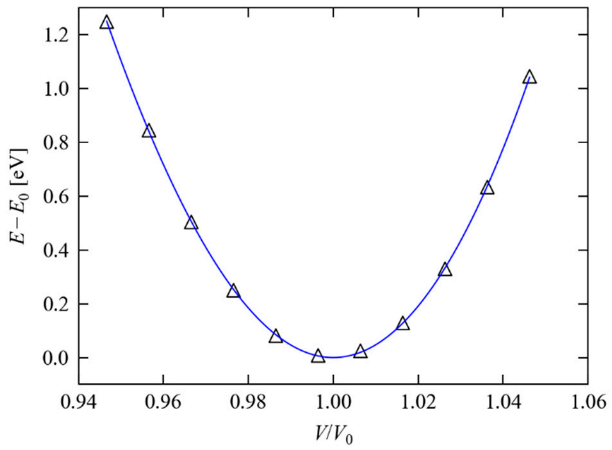

Figure S5: Simulated energy-volume curve for faujasite (FAU) obtained with the PBEsol+D3 functional. The black triangles show the data points and the blue curve shows a fit to the Birch-Murnaghan equation.

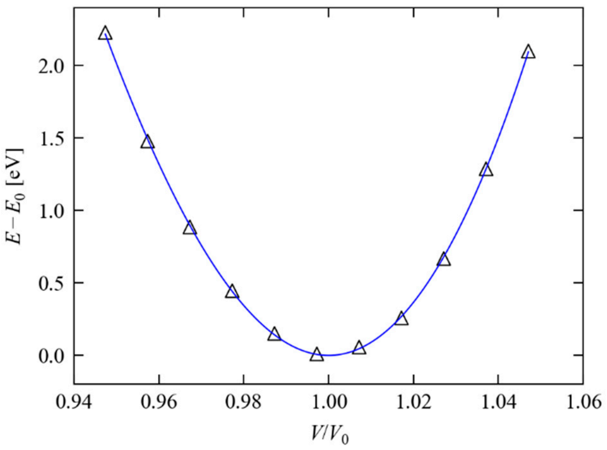

Figure S6: Simulated energy-volume curve for zeolite EMC-2 (EMT) obtained with the PBEsol+D3 functional. The black triangles show the data points and the blue curve shows a fit to the Birch-Murnaghan equation.

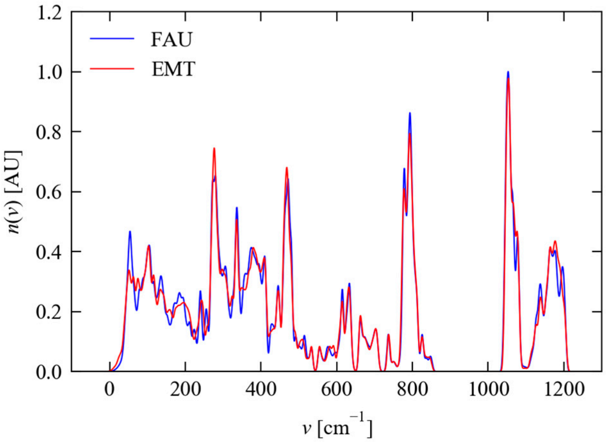

Figure S7: Simulated phonon density of states (DoS) curves of the zeolites faujasite and EMC-2 obtained with the PBEsol+D3 functional. Zeolite faujasite (FAU) is shown in blue, and EMC-2 (EMT) in red.

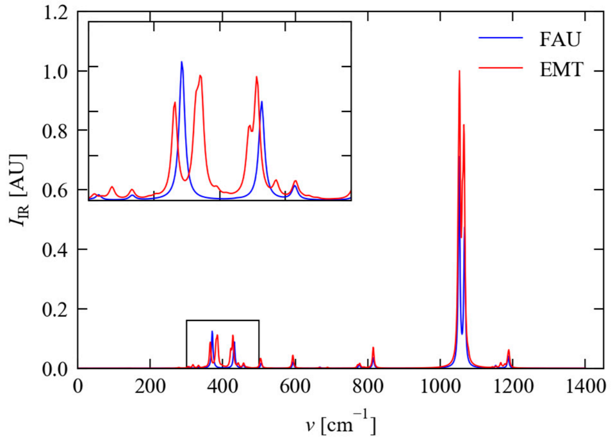

Figure S8: Simulated infrared (IR) spectra of the zeolites faujasite and EMC-2 obtained with the PBEsol+D3 functional. Zeolite faujasite (FAU) is shown in blue, and EMC-2 (EMT) in red. The inset plot shows an expansion of the region from 300-500 cm^{-1} to highlight a group of bands that could potentially be used to distinguish the two structures using IR spectroscopy.

References

62. Yokogawa, K.; Murata, K.; Yoshino, H.; Aoyama, S. Solidification of high-pressure medium Daphne 7373. *Jpn. J. Appl. Phys.* **2007**, *46*, 3636, doi:10.1143/jjap.46.3636.
63. Klotz, S.; Chervin, J.C.; Munsch, P.; Le Marchand, G. Hydrostatic limits of 11 pressure transmitting media. *J. Phys. D: Appl. Phys.* **2009**, *42*, 075413, doi:10.1088/0022-3727/42/7/075413.
64. De Jong, M.; Chen, W.; Angsten, T.; Jain, A.; Notestine, R.; Gamst, A.; Sluiter, M.; Ande, C.K.; Van Der Zwaag, S.; Plata, J.J. Charting the complete elastic properties of inorganic crystalline compounds. *Sci. Data* **2015**, *2*, 150009, doi:10.1038/sdata.2015.9.
